# How do we define and measure sarcopenia? Protocol for a systematic review

**DOI:** 10.1186/s13643-018-0712-y

**Published:** 2018-03-27

**Authors:** Paulo Roberto Carvalho do Nascimento, Stéphane Poitras, Martin Bilodeau

**Affiliations:** 1 School of Rehabilitation Sciences, Faculty of Health Sciences, University of Ottawa, 43 Bruyère Street, Ottawa, ON K1N5C8 Canada; 2 Bruyère Research Institute, Ottawa, ON Canada; 3 School of Human Kinetics, Faculty of Health Sciences, University of Ottawa, Ottawa, ON Canada

**Keywords:** Sarcopenia, Aging, Aged, Bias, Epidemiology

## Abstract

**Background:**

The loss of muscle mass is a natural aging consequence. A reduction of muscle mass that surpasses the physiological rate is considered the key factor responsible for the development of a geriatric syndrome called sarcopenia. However, a new understanding of the importance of muscle quality over quantity is rising; as a result, different definitions for sarcopenia has been used. Due to the negative impact on elder’s health and quality of life, the number of research investigating the causes, prevalence, and management of sarcopenia is increasing, although a consensus on sarcopenia definition is still missing. This systematic review will assess observational studies reporting the presence of sarcopenia aiming to verify how sarcopenia is defined, the diagnosis criteria, and the tools used for assessment. In addition, we will investigate the influence of the definition and diagnostic tools on the prevalence rate.

**Methods:**

Keywords related to the condition, population, and type of study will be combined to build a search strategy for each of the following databases MEDLINE, EMBASE, CINAHL (*Cumulative Index to Nursing and Allied Health Literature*), Web of Science, and Google Scholar. Two independent reviewers will analyze the retrieved papers for eligibility and the methodological quality of eligible studies. The definition of sarcopenia and diagnostic tools used in each study and the prevalence estimates will be extracted. Descriptive statistics will be used to report the definitions of sarcopenia, diagnostic tools, and whether these influence or not, the prevalence rates.

**Discussion:**

Sarcopenia is receiving greater attention in geriatrics research in recent years. Therefore, it is important to investigate how this condition is defined in the literature and whether these definitions can interfere with the reported estimates devoting more efforts on the topic. The results of this study can help to determine the most used definitions of sarcopenia reported in the literature, its strengths and limitations, and open a discussion about a need for a more valid, easy, and suitable one.

**Systematic review registration:**

PROSPERO CRD42015020832

**Electronic supplementary material:**

The online version of this article (10.1186/s13643-018-0712-y) contains supplementary material, which is available to authorized users.

## Background

Sarcopenia is a geriatric syndrome affecting older adults, which was firstly described by Rosenberg [1] as the loss of muscle mass in seniors. In addition to the loss of muscle mass [2, 3], aging is also accompanied by a reduction in muscle strength [4, 5] and decline in physical function [6, 7], which are combined to define sarcopenia according to a contemporary definition [8]. These alterations may be associated with changes in muscular quality [3, 9] due to the reduction in the size [10–12], number [10, 12], and contractility of the muscle fiber [13, 14], as well as fat tissue infiltration in the muscle [15, 16].

Prevalence of sarcopenia increases with age advance [17, 18]. However, it is not possible to rely on this estimates due to the lack of a universal definition of sarcopenia. Despite the effort from the European Working Group on Sarcopenia in Older People (EWGSOP) [8] to diagnose sarcopenia, results from two recent systematic reviews [19, 20] pooling the prevalence estimates for sarcopenia presented discrepant values of 10 and 29%, respectively. The difference in the results of these reviews seems to be due to the lack of similarity in defining sarcopenia.

A clear definition of sarcopenia is important since the number of publications on this syndrome is increasing [19, 21–24], and especially due to the fact that sarcopenia is associated with an increased risk for all-cause of mortality (OR = 3.64, 95% CI = 2.94 to 4.51) and functional decline (OR = 2.58, 95% CI = 1.33 to 4.99) [24], summed to a high economic cost [25]. Considering that older adults are a growing population group around the world [26], the burden due sarcopenia tends to be higher. The negative consequences resulted from sarcopenia have stimulated the development of studies about its prevalence [19, 20] and management [20]. However, to date, no study has comprehensively evaluated the definitions and tools used in the literature to define and determine the presence of sarcopenia.

The lack of a consensus on defining sarcopenia prevents estimating the prevalence and prognosis and comparing the effectiveness of interventions between clinical trials. The EWGSOP [8] was the first group attempting to provide a consensus definition for sarcopenia followed by the International Working Group on Sarcopenia (IWGS) [27], and by the Asian Working Group for Sarcopenia (AWGS) [28], respectively. These groups defined sarcopenia based on the appendicular muscle mass adjusted by the height squared, the handgrip strength, and/or gait speed presenting a certain variation from each other. Further, an initiative from the Foundation for the National Institutes of Health (FNIH) proposed that sarcopenia should be defined based on muscle mass adjusted by the body mass index (BMI) with cutoff values of (< 0.789 kg/m^2^ men and < 0.512 kg/m^2^ women) and grip strength (< 26 kg men and < 16 kg women) [29].

Although the definitions provided by the EWGSOP, IWGS, AWGS, and FNIH use different strategies and cutoff points to normalize and define loss of muscle mass, reduction in muscle strength, and low gait speed, the loss of muscle mass is considered the starting point for the development of sarcopenia. However, recent studies provided information that reduction in muscle quality surpass the loss of muscle mass and that the aging decline is respectively greater in muscle power, strength, and mass [30–32]. Furthermore, in a recent study, dos Santos et al. [33] observed that in a population of older adults (+ 90 years old), participants with low muscle mass had 1.65 (95% CI 1.27–2.31) increased odds for being at risk for losing physical independence and participants with low muscle force had 6.19 (95% CI 5.08–7.53) increased odds for being at risk of losing physical independence. As a result, new views on the criterion to define sarcopenia are emerging, not limiting sarcopenia to loss of muscle mass but, instead, as a loss of muscle strength due to alterations in the muscle quality related to the age advance [34]. Nevertheless, it is unclear whether these definitions are used in research reporting sarcopenia estimates and how they influence the results. Thus, the primary aim of this systematic review will be to identify how sarcopenia is defined and measured in the literature reporting its prevalence. Secondly, we will evaluate how different definitions can affect prevalence estimates.

## Methods

### Protocol and registration

This protocol is reported following the Preferred Reporting Items for Systematic Reviews and Meta-Analyses Protocols (PRISMA-P checklist) [35] according to the elaboration and explanation guideline [36]. The PRISMA-P checklist is included as an additional file [see Additional file 1]. This protocol is registered with PROSPERO no. CRD42015020832.

### Selection criteria

All indexed observational population-based studies published, in which the prevalence of sarcopenia in community-dwelling older adults was reported, will be considered for review independent of the language of publication and publication date.

### Exclusion criteria

We will exclude articles reporting on the prevalence of sarcopenia in participants with specific health issues (e.g., diabetes, cancer, and organ transplantation). Furthermore, we will not include articles written in languages other than English, French, or Portuguese, which could not be translated by the authors.

### Search strategy

An electronic search will be conducted using the databases MEDLINE, EMBASE, CINAHL (*Cumulative Index to Nursing and Allied Health Literature*), Web of Science (Core Collection), and Google Scholar. A search strategy was built for each database (Appendix 1) using a combination of specific terms for (a) target population—“elderly,” “older adults,” “older people,” “older person,” and “community-dwelling;” (b) condition—“sarcopenia,” “aging,” and “muscular atrophy;” and (c) type of study—“prevalence,” “incidence,” “epidemiology,” “cross-sectional,” and “cohort studies.” We will include studies that analyzed prevalence of sarcopenia published in peer-reviewed journals through February 2018.

Additionally, we will perform a comprehensive examination of reference lists from eligible studies. Data pertaining to individuals under 60 years old will not be considered.

All retrieved papers will be exported to a reference manager software (Endnote®), then examined by two independent researchers (PN an MB), through the readings of the title, abstract, and full text. In each stage, studies that do not fulfill eligibility criteria will be excluded. In cases of disagreements between reviewers that cannot be resolved by consensus, a third opinion will be consulted for final arbitration.

### Quality appraisal

Two independent researchers (PN and MB) will critically appraise the quality of each eligible study using the quality assessment tool for observational cohort and cross-sectional studies proposed by the National Heart, Lung and Blood Institute (Appendix 2).

The quality will be based on the following items: (1) clear question and objective, (2) target population, (3) participation rate, (4) sample selection, (5) sample size justification, (6) temporal relationship for exposure/outcome, (7) length of the timeframe, (8) levels of the exposure of interest, (9) exposure measure details, (10) number of exposure measurements, (11) outcome measures, (12) blinding of outcome assessors, (13) follow-up rate, and (14) statistical analyses.

### Data extraction and analysis

The information about the articles (author, type of study, data collection strategy, sample size, age, gender, definition of sarcopenia, measurement tools, and prevalence rate) will be extracted independently by two researchers (PN and MB) using an electronic sheet.

Frequency distribution will be used to present the definitions and tools used to diagnose sarcopenia through the studies. We will analyze the influence of the definitions of sarcopenia on prevalence estimates according to mean age or age strata (i.e., 60–70 years, 71–80 years, > 80 years), presenting results using descriptive statistics.

## Discussion

To our knowledge, this will be the first systematic review analyzing the definitions and tools used to diagnose sarcopenia. This review will provide a summary of the sarcopenia definitions currently used to diagnose sarcopenia and implications in terms of estimates. Further, it provides evidence for discussion on how to best define sarcopenia.

A standard definition and screening tools for sarcopenia are important to provide valid and reproducible values allowing reliable measures and comparison between estimates. Considering the amount of time and resources expended recently with research on sarcopenia, maybe it is time to take a step back and analyze how well this condition is being diagnosed, the validity and probabilities of false positive or false negative cases provided by the current definitions before applying more efforts with new researches. The results of the systematic review will be presented in scientific events and published in a peer-reviewed journal.

### Additional file

Additional file 1: PRISMA-P checklist. (DOCX 30 kb)

## Appendix 1

**Table 1:** Search strategy

Web of Science	Google Scholar	MEDLINE	EMBASE	CINAHL
# 1 TS = (sarcopen* OR aging OR muscle atrophy) # 2 TI = (prevalence OR incidence OR cross-sectional OR cohort) # 3 TI = (older adults OR elder* OR seniors OR community-dwelling) (#3 AND #2 AND #1)	Sarcopenia prevalence Prevalence of sarcopenia	1. Sarcopenia/ 2. sarcopenia.ti,ab. 3. 1 or 2 4. Muscular Atrophy/ 5. (musc* adj1 atrophy).ti,ab. 6. 4 or 5 7. aging.ti,ab. 8. Aging/ 9. 7 or 8 10. 6 and 9 11. “Aged, 80 and over”/ or Aged/ 12. Prevalence/ 13. Cross-Sectional Studies/ 14. 12 or 13 15. 3 or 10 16. 11 and 14 and 15	1. Sarcopenia/ 2. sarcopenia.ti,ab. 3. 1 or 2 4. Muscular Atrophy/ 5. (musc* adj1 atrophy).ti,ab. 6. 4 or 5 7. Aging/ 8. aged.ti,ab. 9. 7 or 8 10. 6 and 9 11. Aged/ 12. “Aged, 80 and over”/ 13. 11 or 12 14. Prevalence/ 15. Incidence/ 16. Cohort Studies/ 17. Cross-Sectional Studies/ 18. 3 or 10 19. 14 or 15 or 16 or 17 20. 13 and 18 and 19	S1. TI Sarcopenia OR AB Sarcopenia S2. TI (MH “Muscular Atrophy”) S3. S1 OR S2 S4. TI Aged OR AB Aged S5. (MH “Aged, 80 and Over”) S6. S4 OR S5 S7. (MH “Prevalence”) OR (MH “Cross Sectional Studies”) OR (MH “Surveys”) OR (MH “Epidemiological Research”) S8. S3 AND S6 AND S7

## Appendix 2

**Table:**

Criteria	Yes	No	Other (CD, NR, NA)
1. Was the research question or objective in this paper clearly stated?			
2. Was the study population clearly specified and defined?			
3. Was the participation rate of eligible persons at least 50%?			
4. Were all the subjects selected or recruited from the same or similar populations (including the same time period)? Were inclusion and exclusion criteria for being in the study prespecified and applied uniformly to all participants?			
5. Was a sample size justification, power description, or variance and effect estimates provided?			
6. For the analyses in this paper, were the exposure(s) of interest measured prior to the outcome(s) being measured?			
7. Was the timeframe sufficient so that one could reasonably expect to see an association between exposure and outcome if it existed?			
8. For exposures that can vary in amount or level, did the study examine different levels of the exposure as related to the outcome (e.g., categories of exposure, or exposure measured as continuous variable)?			
9. Were the exposure measures (independent variables) clearly defined, valid, reliable, and implemented consistently across all study participants?			
10. Was the exposure(s) assessed more than once over time?			
11. Were the outcome measures (dependent variables) clearly defined, valid, reliable, and implemented consistently across all study participants?			
12. Were the outcome assessors blinded to the exposure status of participants?			
13. Was loss to follow-up after baseline 20% or less?			
14. Were key potential confounding variables measured and adjusted statistically for their impact on the relationship between exposure(s) and outcome(s)?			
Quality rating (Good, Fair, or Poor) (see guidance)			
Rater #1 initials:			
Rater #2 initials:			
Additional comments (If Poor, please state why):			

*CD* cannot determine, *NA* not applicable, *NR* not reported

### Guidance for assessing the quality of observational cohort and cross-sectional studies

The guidance document below is organized by question number from the tool for quality assessment of observational cohort and cross-sectional studies.

#### Question 1. Research question

Did the authors describe their goal in conducting this research? Is it easy to understand what they were looking to find? This issue is important for any scientific paper of any type. Higher quality scientific research explicitly defines a research question.

#### Questions 2 and 3. Study population

Did the authors describe the group of people from which the study participants were selected or recruited, using demographics, location, and time period? If you were to conduct this study again, would you know who to recruit, from where, and from what time period? Is the cohort population free of the outcomes of interest at the time they were recruited?

An example would be men over 40 years old with type 2 diabetes who began seeking medical care at Phoenix Good Samaritan Hospital between January 1, 1990, and December 31, 1994. In this example, the population is clearly described as (1) who (men over 40 years old with type 2 diabetes), (2) where (Phoenix Good Samaritan Hospital), and (3) when (between January 1, 1990, and December 31, 1994). Another example is women ages 34 to 59 years of age in 1980 who were in the nursing profession and had no known coronary disease, stroke, cancer, hypercholesterolemia, or diabetes, and were recruited from the 11 most populous States, with contact information obtained from State nursing boards.

In cohort studies, it is crucial that the population at baseline is free of the outcome of interest. For example, the nurses’ population above would be an appropriate group in which to study incident coronary disease. This information is usually found in either descriptions of population recruitment, definitions of variables, or inclusion/exclusion criteria.

You may need to look at prior papers on methods in order to make the assessment for this question. Those papers are usually in the reference list.

If fewer than 50% of eligible persons participated in the study, then there is concern that the study population does not adequately represent the target population. This increases the risk of bias.

#### Question 4. Groups recruited from the same population and uniform eligibility criteria

Were the inclusion and exclusion criteria developed prior to recruitment or selection of the study population? Were the same underlying criteria used for all of the subjects involved? This issue is related to the description of the study population, above, and you may find the information for both of these questions in the same section of the paper.

Most cohort studies begin with the selection of the cohort; participants in this cohort are then measured or evaluated to determine their exposure status. However, some cohort studies may recruit or select exposed participants in a different time or place than unexposed participants, especially retrospective cohort studies—which is when data are obtained from the past (retrospectively), but the analysis examines exposures prior to outcomes. For example, one research question could be whether diabetic men with clinical depression are at higher risk for cardiovascular disease than those without clinical depression. So, diabetic men with depression might be selected from a mental health clinic, while diabetic men without depression might be selected from an internal medicine or endocrinology clinic. This study recruits groups from different clinic populations, so this example would get a “no.”

However, the women nurses described in the question above were selected based on the same inclusion/exclusion criteria, so that example would get a “yes.”

#### Question 5. Sample size justification

Did the authors present their reasons for selecting or recruiting the number of people included or analyzed? Do they note or discuss the statistical power of the study? This question is about whether or not the study had enough participants to detect an association if one truly existed.

A paragraph in the methods section of the article may explain the sample size needed to detect a hypothesized difference in outcomes. You may also find a discussion of power in the discussion section (such as the study had 85% power to detect a 20% increase in the rate of an outcome of interest, with a two-sided alpha of 0.05). Sometimes estimates of variance and/or estimates of effect size are given, instead of sample size calculations. In any of these cases, the answer would be “yes.”

However, observational cohort studies often do not report anything about power or sample sizes because the analyses are exploratory in nature. In this case, the answer would be “no.” This is not a “fatal flaw.” It just may indicate that attention was not paid to whether the study was sufficiently sized to answer a prespecified question—i.e., it may have been an exploratory, hypothesis-generating study.

#### Question 6. Exposure assessed prior to outcome measurement

This question is important because, in order to determine whether an exposure causes an outcome, the exposure must come before the outcome.

For some prospective cohort studies, the investigator enrolls the cohort and then determines the exposure status of various members of the cohort (large epidemiological studies like Framingham used this approach). However, for other cohort studies, the cohort is selected based on its exposure status, as in the example above of depressed diabetic men (the exposure being depression). Other examples include a cohort identified by its exposure to fluoridated drinking water and then compared to a cohort living in an area without fluoridated water, or a cohort of military personnel exposed to combat in the Gulf War compared to a cohort of military personnel not deployed in a combat zone.

With either of these types of cohort studies, the cohort is followed forward in time (i.e., prospectively) to assess the outcomes that occurred in the exposed members compared to non-exposed members of the cohort. Therefore, you begin the study in the present by looking at groups that were exposed (or not) to some biological or behavioral factor, intervention, etc., and then you follow them forward in time to examine outcomes. If a cohort study is conducted properly, the answer to this question should be “yes,” since the exposure status of members of the cohort was determined at the beginning of the study before the outcomes occurred.

For retrospective cohort studies, the same principal applies. The difference is that, rather than identifying a cohort in the present and following them forward in time, the investigators go back in time (i.e., retrospectively) and select a cohort based on their exposure status in the past and then follow them forward to assess the outcomes that occurred in the exposed and non-exposed cohort members. Because in retrospective cohort studies the exposure and outcomes may have already occurred (it depends on how long they follow the cohort), it is important to make sure that the exposure preceded the outcome.

Sometimes cross-sectional studies are conducted (or cross-sectional analyses of cohort study data), where the exposures and outcomes are measured during the same timeframe. As a result, cross-sectional analyses provide weaker evidence than regular cohort studies regarding a potential causal relationship between exposures and outcomes. For cross-sectional analyses, the answer to Question 6 should be “no.”

#### Question 7. Sufficient timeframe to see an effect

Did the study allow enough time for a sufficient number of outcomes to occur or be observed, or enough time for an exposure to have a biological effect on an outcome? In the examples given above, if clinical depression has a biological effect on increasing risk for CVD, such an effect may take years. In the other example, if higher dietary sodium increases BP, a short timeframe may be sufficient to assess its association with BP, but a longer timeframe would be needed to examine its association with heart attacks.

The issue of timeframe is important to enable meaningful analysis of the relationships between exposures and outcomes to be conducted. This often requires at least several years, especially when looking at health outcomes, but it depends on the research question and outcomes being examined.

Cross-sectional analyses allow no time to see an effect, since the exposures and outcomes are assessed at the same time, so those would get a “no” response.

#### Question 8. Different levels of the exposure of interest

If the exposure can be defined as a range (examples: drug dosage, amount of physical activity, amount of sodium consumed), were multiple categories of that exposure assessed? (for example, for drugs: not on the medication, on a low dose, medium dose, high dose; for dietary sodium, higher than average US consumption, lower than recommended consumption, between the two). Sometimes discrete categories of exposure are not used, but instead exposures are measured as continuous variables (for example, mg/day of dietary sodium or BP values).

In any case, studying different levels of exposure (where possible) enables investigators to assess trends or dose-response relationships between exposures and outcomes—e.g., the higher the exposure, the greater the rate of the health outcome. The presence of trends or dose-response relationships lends credibility to the hypothesis of causality between exposure and outcome.

For some exposures, however, this question may not be applicable (e.g., the exposure may be a dichotomous variable like living in a rural setting versus an urban setting, or vaccinated/not vaccinated with a one-time vaccine). If there are only two possible exposures (yes/no), then this question should be given an “NA,” and it should not count negatively towards the quality rating.

#### Question 9. Exposure measures and assessment

Were the exposure measures defined in detail? Were the tools or methods used to measure exposure accurate and reliable—for example, have they been validated or are they objective? This issue is important as it influences confidence in the reported exposures. When exposures are measured with less accuracy or validity, it is harder to see an association between exposure and outcome even if one exists. Also as important is whether the exposures were assessed in the same manner within groups and between groups; if not, bias may result.

For example, retrospective self-report of dietary salt intake is not as valid and reliable as prospectively using a standardized dietary log plus testing participants’ urine for sodium content. Another example is measurement of BP, where there may be quite a difference between usual care, where clinicians measure BP however it is done in their practice setting (which can vary considerably), and use of trained BP assessors using standardized equipment (e.g., the same BP device which has been tested and calibrated) and a standardized protocol (e.g., patient is seated for 5 min with feet flat on the floor, BP is taken twice in each arm, and all four measurements are averaged). In each of these cases, the former would get a “no” and the latter a “yes.”

Here is a final example that illustrates the point about why it is important to assess exposures consistently across all groups: If people with higher BP (exposed cohort) are seen by their providers more frequently than those without elevated BP (non-exposed group), it also increases the chances of detecting and documenting changes in health outcomes, including CVD-related events. Therefore, it may lead to the conclusion that higher BP leads to more CVD events. This may be true, but it could also be due to the fact that the subjects with higher BP were seen more often; thus, more CVD-related events were detected and documented simply because they had more encounters with the health care system. Thus, it could bias the results and lead to an erroneous conclusion.

#### Question 10. Repeated exposure assessment

Was the exposure for each person measured more than once during the course of the study period? Multiple measurements with the same result increase our confidence that the exposure status was correctly classified. Also, multiple measurements enable investigators to look at changes in exposure over time, for example, people who ate high dietary sodium throughout the follow-up period, compared to those who started out high then reduced their intake, compared to those who ate low sodium throughout. Once again, this may not be applicable in all cases. In many older studies, exposure was measured only at baseline. However, multiple exposure measurements do result in a stronger study design.

#### Question 11. Outcome measures

Were the outcomes defined in detail? Were the tools or methods for measuring outcomes accurate and reliable—for example, have they been validated or are they objective? This issue is important because it influences confidence in the validity of study results. Also important is whether the outcomes were assessed in the same manner within groups and between groups.

An example of an outcome measure that is objective, accurate, and reliable is death—the outcome measured with more accuracy than any other. But even with a measure as objective as death, there can be differences in the accuracy and reliability of how death was assessed by the investigators. Did they base it on an autopsy report, death certificate, death registry, or report from a family member? Another example is a study of whether dietary fat intake is related to blood cholesterol level (cholesterol level being the outcome), and the cholesterol level is measured from fasting blood samples that are all sent to the same laboratory. These examples would get a “yes.” An example of a “no” would be self-report by subjects that they had a heart attack, or self-report of how much they weigh (if body weight is the outcome of interest).

Similar to the example in Question 9, results may be biased if one group (e.g., people with high BP) is seen more frequently than another group (people with normal BP) because more frequent encounters with the health care system increase the chances of outcomes being detected and documented.

#### Question 12. Blinding of outcome assessors

Blinding means that outcome assessors did not know whether the participant was exposed or unexposed. It is also sometimes called “masking.” The objective is to look for evidence in the article that the person(s) assessing the outcome(s) for the study (for example, examining medical records to determine the outcomes that occurred in the exposed and comparison groups) is masked to the exposure status of the participant. Sometimes the person measuring the exposure is the same person conducting the outcome assessment. In this case, the outcome assessor would most likely not be blinded to exposure status because they also took measurements of exposures. If so, make a note of that in the comments section.

As you assess this criterion, think about whether it is likely that the person(s) doing the outcome assessment would know (or be able to figure out) the exposure status of the study participants. If the answer is no, then blinding is adequate. An example of adequate blinding of the outcome assessors is to create a separate committee, whose members were not involved in the care of the patient and had no information about the study participants’ exposure status. The committee would then be provided with copies of participants’ medical records, which had been stripped of any potential exposure information or personally identifiable information. The committee would then review the records for prespecified outcomes according to the study protocol. If blinding was not possible, which is sometimes the case, mark “NA” and explain the potential for bias.

#### Question 13. Follow-up rate

Higher overall follow-up rates are always better than lower follow-up rates, even though higher rates are expected in shorter studies, whereas lower overall follow-up rates are often seen in studies of longer duration. Usually, an acceptable overall follow-up rate is considered 80% or more of participants whose exposures were measured at baseline. However, this is just a general guideline. For example, a 6-month cohort study examining the relationship between dietary sodium intake and BP level may have over 90% follow-up, but a 20-year cohort study examining effects of sodium intake on stroke may have only a 65% follow-up rate.

#### Question 14. Statistical analyses

Were key potential confounding variables measured and adjusted for, such as by statistical adjustment for baseline differences? Logistic regression or other regression methods are often used to account for the influence of variables not of interest.

This is a key issue in cohort studies, because statistical analyses need to control for potential confounders, in contrast to an RCT, where the randomization process controls for potential confounders. All key factors that may be associated both with the exposure of interest and the outcome—that are not of interest to the research question—should be controlled for in the analyses.

For example, in a study of the relationship between cardiorespiratory fitness and CVD events (heart attacks and strokes), the study should control for age, BP, blood cholesterol, and body weight, because all of these factors are associated both with low fitness and with CVD events. Well-done cohort studies control for multiple potential confounders.

### Some general guidance for determining the overall quality rating of observational cohort and cross-sectional studies

The questions on the form are designed to help you focus on the key concepts for evaluating the internal validity of a study. They are not intended to create a list that you simply tally up to arrive at a summary judgment of quality.

Internal validity for cohort studies is the extent to which the results reported in the study can truly be attributed to the exposure being evaluated and not to flaws in the design or conduct of the study—in other words, the ability of the study to draw associative conclusions about the effects of the exposures being studied on outcomes. Any such flaws can increase the risk of bias.

Critical appraisal involves considering the risk of potential for selection bias, information bias, measurement bias, or confounding (the mixture of exposures that one cannot tease out from each other). Examples of confounding include co-interventions, differences at baseline in patient characteristics, and other issues throughout the questions above. High risk of bias translates to a rating of poor quality. Low risk of bias translates to a rating of good quality. (Thus, the greater the risk of bias, the lower the quality rating of the study).

In addition, the more attention in the study design to issues that can help determine whether there is a causal relationship between the exposure and outcome, the higher quality the study. These include exposures occurring prior to outcomes, evaluation of a dose-response gradient, accuracy of measurement of both exposure and outcome, sufficient timeframe to see an effect, and appropriate control for confounding—all concepts reflected in the tool.

Generally, when you evaluate a study, you will not see a “fatal flaw,” but you will find some risk of bias. By focusing on the concepts underlying the questions in the quality assessment tool, you should ask yourself about the potential for bias in the study you are critically appraising. For any box where you check “no” you should ask, “What is the potential risk of bias resulting from this flaw in study design or execution?” That is, does this factor cause you to doubt the results that are reported in the study or doubt the ability of the study to accurately assess an association between exposure and outcome?

The best approach is to think about the questions in the tool and how each one tells you something about the potential for bias in a study. The more you familiarize yourself with the key concepts, the more comfortable you will be with critical appraisal. Examples of studies rated good, fair, and poor are useful, but each study must be assessed on its own based on the details that are reported and consideration of the concepts for minimizing bias.

## Abbreviations

AWGS: Asian Working Group for Sarcopenia
BMI: Body mass index
CINAHL: *Cumulative Index to Nursing and Allied Health Literature*
EWGSOP: European Working Group on Sarcopenia in Older People
FNIH: Foundation for the National Institutes of Health
IWGS: International Working Group on Sarcopenia
PRISMA-P: Preferred Reporting Items for Systematic Reviews and Meta-Analyses Protocols
